# Transforming Musculoskeletal Care: Effectiveness of a Physician-Led Telemedicine Integrated Practice Unit

**DOI:** 10.1177/26924366251382437

**Published:** 2025-09-25

**Authors:** Ryan A. Grant, Mary I. O’Connor, Patrick Myers, Jim Fiechtl

**Affiliations:** Vori Health, Nashville, Tennessee, USA.

**Keywords:** musculoskeletal, integrated practice unit, digital care, telemedicine

## Abstract

**Background:**

The growing crisis of musculoskeletal (MSK) disease is overwhelming health care systems and straining payors worldwide. Current telemedicine programs that focus solely on exercise therapy are not structured to deliver comprehensive care. In contrast, physician-led integrated practice units (IPUs)—multidisciplinary medical teams designed around patient-centered, holistic care—offer an innovative path forward.

**Methods:**

We analyzed the clinical outcomes of 2,851 patients treated in our digital MSK IPU.

**Results:**

Pain (scale, 0–10) improved in 75.2% of all patients from 4.7 to 2.6 and in 84.2% in those with severe pain (≥7) from 7.7 to 4.1. Functional status (Single assessment numeric evaluation [SANE] scale, 0–100) in 2,018 patients, significantly improved in 68.9% from 54.6 to 73.7. Even more compelling, in 274 patients with severe functional compromise (SANE ≤20), scores improved 599.0% from 7.9 to 55.1.

**Conclusion:**

Our results underscore the power of a telemedicine-based IPU to deliver significant reductions in pain and dramatic functional gains, even for patients starting with severe limitations. As the demand for MSK care continues to surge, a digital MSK IPU is a promising model to support both access to care and quality clinical outcomes.

## Introduction

National health care expenditure continues to escalate and is estimated to be 20.3% of the gross domestic product by 2033.^1^ Chronic musculoskeletal (MSK) pain and disability represent a significant and increasing proportion of health care expenditure. From 2000 to 2021, the prevalence of MSK disease increased by nearly 22% in the United States, including a 114% increase in osteoarthritis in adults.^2^ For aggregated conditions, MSK health care spend is surpassed only by cardiovascular diseases and neoplasms.^3^ With the aging of the population further increasing the burden of MSK disease, innovating care for these patients to be effective and value driven is essential for the health of both the nation and the health care delivery system.

Traditional care models of primary care clinicians, physical therapists, and orthopedic and spine surgeons typically produce fragmented care for patients,^2^ contributing to overutilization of costly advanced imaging^4–7^ and inappropriate surgeries.^8–10^ Innovations in the delivery of MSK care include integrated practice units (IPU), defined as a multidisciplinary team of health care professions organized around the treatment of a specific condition or related conditions and designed for more comprehensive and holistic care of the patient,^11–13^ as well as digital care services offering greater access to care, convenience for individuals, and lower cost to care delivery.

Digital MSK interventions can vary from fully self-directed by the individual to telerehabilitation directed by a physical therapist to telemedicine services provided by a physician. While numerous published studies show evidence that digital self-directed programs and telerehabilitation directed by a physical therapist can improve function and pain in participants,^14–16^ the heterogenicity of study populations relative to the prior medical evaluation and treatment of their MSK concern limit an understanding of what type of digital offering is appropriate for an individual at a given time. Specifically digital self-directed programs and digital telerehabilitation by a physical therapist cannot provide patients with a medical diagnosis, appropriate ordering of medications and imaging, and integrated multidisciplinary care. Such models are not designed to address the complex, multifaceted nature of chronic MSK conditions. Rather innovative, integrated care approaches are needed to deliver superior clinical outcomes and restore meaningful function particularly in patients with more severe pain and disability.

Against this backdrop, our study rigorously evaluates the transformative impact of a digital physician-led MSK IPU on pain, functional, and mental health outcomes in a large and diverse cohort of patients exhibiting a broad spectrum of MSK dysfunction, including those initially classified as fully disabled based on patient reported functional measures. While in-person MSK IPUs have demonstrated effective outcomes,^13,17,18^ we are unaware of prior publications related to a digital MSK IPU for the broad spectrum of MSK conditions.

## Methods

Our study was deemed exempt by the Institutional Board Review. To ensure the integrity and reliability of the study, several inclusion and exclusion criteria were applied. The study included adults aged 18 years or older with MSK symptoms who presented to us for evaluation and met the required number of clinical visits (detailed below). No clinical conditions were excluded from this study. Adults who were eligible for accessing our medical care, and thus to potentially become study participants, were those with insurance benefits which included our services. Patients could self-schedule an evaluation with us or be referred to us by another clinician.

Patients were excluded from this study who were inappropriate for digital care. This determination was made at the time of the initial digital evaluation and included patients who were unable to independently use technology for telehealth, had cognitive or communication impairment preventing effective engagement with digital therapy, and lacked access to a safe environment for digital care; these patients were discharged from our practice. Additional exclusion criteria included patients with conditions requiring hands-on physical therapy techniques (e.g., manual therapy for severe joint restrictions or balance dysfunction with risk of falling); such patients represent <5% of patients evaluated in our practice. Patients who required referral for in-person surgical consultation were included in the study if they met the inclusion criteria.

Patients were required to have completed a combined initial evaluation between 9.1.2023 and 2.10.2025 with both a physician or nurse practitioner (rendering the medical diagnosis) and a physical therapist (rendering the functional diagnosis) during the same simultaneous digital encounter.^19^ Patients who started with a single clinician or on their own (self-management) were excluded. Thereafter, a minimum of two subsequent clinical visits were required for inclusion. Follow-up visits were typically with physical therapists or physical therapy assistants. If clinically appropriate, patients returned for further evaluation by the physician or nurse practitioner. Patients who had concerning scores on the Generalized Anxiety Disorder Scale-2 (GAD-2) and/or Patient Health Questionnaire-2 (PHQ-2) surveys, namely scores of 3 or higher on either survey, had more intensive cognitive behavioral therapy infused into their care plans. Health coaches also supported patients with lifestyle improvement and registered dietitians addressed patients with obesity and nutritional challenges. All clinical visits were on our digital health platform.

If clinically appropriate, patients were referred for imaging studies, injections, surgical consultation, or advanced mental health care typically under the direction of the patient’s primary care physician. These patients were included in this study if they met the required three clinical visit requirement.

Baseline and follow-up patient data collected included pain scores (numeric pain rating scale),^20^ Single Assessment Numeric Evaluation (SANE) scores,^21^ and patient-reported mental health questionnaires, namely the GAD-2^22^ and the PHQ-2.^23^

The SANE score is a patient reported measure in which the patient answers the following question: “How would you rate your [affected body part] today as a percentage of normal, where 0% is completely abnormal and 100% is normal?”^24^ Most patients presenting for treatment of MSK symptoms have SANE scores in the mid 50s.^25^ A SANE score of 40 or lower reflects significant functional limitation, often indicative of profound impairment in the ability to perform daily activities, work, or engage in physical tasks.

The GAD-2 questionnaire is a screening tool for anxiety with a range of scores from 0–6, with 3 indicating a positive screen for generalized anxiety disorder. The PHQ-2 questionnaire is a screening tool for depression with a range of score from 0–6, with 3 indicating a positive screen and high likelihood of a major depressive disorder. While we currently have changed our survey protocols, at the time of this study, we did not employ more detailed questionnaires such as GAD-7 or PHQ-9 for patients with a positive screen due to concern regarding survey fatigue related to answering multiple questionnaires.

Medical questionnaire data were excluded if there was no pre- and post-intervention value or if the time between the pre- and post-questionnaires was less than 3 days, to ensure the validity and accuracy of the data in reflecting changes in patients’ conditions over time. These stringent criteria ensured that the sample included only those who met all necessary conditions for digital therapy participation and whose data accurately reflected changes due to the intervention.

### Statistical analysis

This study utilized a rigorous quantitative, retrospective design to evaluate changes in clinical outcomes and to examine associations among key health-related variables. All statistical analyses were conducted using IBM SPSS Statistics, Version 29 (IBM Corp., Armonk, NY). Descriptive statistics were calculated to summarize baseline demographics and clinical measures.

To assess within-subject changes pre- and post-intervention, two-tailed paired *t*-tests were employed with statistical significance set at *p* < 0.05. The distribution of continuous variables was evaluated using the Kolmogorov–Smirnov test for larger samples and the Shapiro–Wilk test for smaller samples. If either test indicated a significant deviation from normality (*p* < 0.05), non-parametric methods were applied to ensure analytical robustness and validity.

Given the potential for non-normal distributions in real-world clinical data, Spearman’s rank-order correlation coefficients were used to assess the strength and direction of monotonic relationships among continuous and ordinal variables, such as pain intensity, functional capacity, and patient-reported outcomes. This approach provided a reliable measure of association without the constraints of normality assumptions.

To compare potential changes across multiple body regions, such as pain scores or functional scores, Kruskal–Wallis tests were employed as appropriate. The Kruskal–Wallis test is a non-parametric alternative to one-way analysis of variance that determines whether there are statistically significant differences in the distribution of a continuous or ordinal variable across two or more independent groups. This test was selected to account for the ordinal nature of certain outcomes and the potential for skewed distributions within subgroup comparisons. When global tests approached statistical significance, post hoc pairwise comparisons were conducted to explore specific group differences. Bonferroni adjustments were applied to control for multiple comparisons and reduce the risk of type I error.

Linear regression analyses were conducted only if the correlation coefficient (ρ or r) exceeded 0.3, signifying at least a moderate association. Clinically and statistically relevant predictors were further explored through multivariate general linear models. In these models, age was treated as a fixed factor and gender as a covariate to adjust for potential confounders. Model assumptions—including linearity, homogeneity of variance-covariance matrices, and multicollinearity—were thoroughly evaluated to ensure model integrity. Effect sizes and 95% confidence intervals were reported to support the interpretation of both statistical and clinical significance. Standard errors of the mean are reported throughout.

## Results

### Demographic, clinical and engagement data

The study cohort comprised 2,851 participants, with patient demographic data, duration of symptoms prior to initial evaluation, completed clinical visits by type of clinician, and duration of digital care shown in Table 1.

**Table 1.** Population Characteristics (n, 2,851)

Age (years)	
Mean	57.8
Median	60
Range	19–97
Sex, *n* (%)	
Female	1,601 (56.16)
Male	1,224 (42.93)
Chose not to disclose or identified as Other	26 (0.91)
Ethnicity, *n* (%)	
Non-Hispanic	1015 (35.6)
Hispanic	80 (2.8)
Chose not to disclose	90 (3.16)
No response	1,666 (58.44)
Race, *n* (%)	
American Indian or Alaskan Native	6 (0.21)
Asian	54 (1.89)
Black	71 (2.49)
White	1,006 (35.29)
Other	41 (1.44)
Chose not to disclose	59 (2.07)
No response	1,614 (56.61)
Symptom duration prior to presentation	
≤6 weeks	541
>6 weeks–3 months	92
>3 months	2,134
Incomplete data	84
Completed clinical visits by type, mean (range)	
MD/NP	1.34 (1–13)
PT/PTA follow-up	4.86 (0–33)
Health coach	1.03 (0–35)
Registered dietitian	0.29 (0–24)
Duration of digital care	
≤14 days	272
15–30 days	603
31–60 days	839
61–90 days	405
91–180 days	448
>180 days	284

MD, Medical Doctor; NP, nurse practitioner; PT, physical therapist; PTA, physical therapist assistant.

**FIG. 1.**
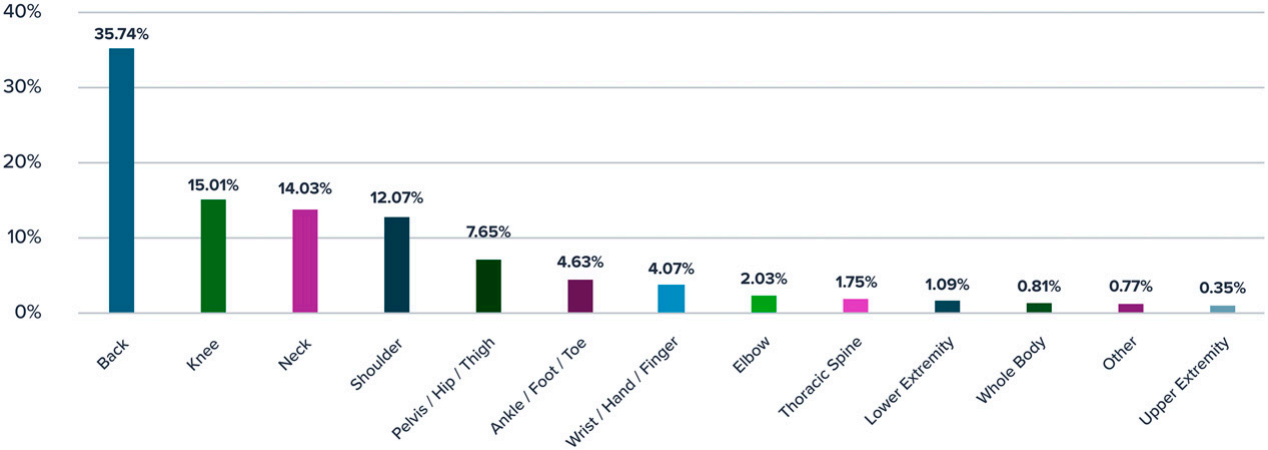
Body region of the primary diagnosis.

Clinical conditions were categorized by affected body part. Back pain was the most common diagnosis, followed by knee pain, neck pain and shoulder pain (Fig. 1). The variation in the number of visits reflects the diverse levels of the underling diagnosis with severe dysfunction requiring more multidisciplinary care.

This comprehensive demographic, clinical, and engagement data provide a detailed context for understanding the diversity and severity of conditions within the study population, setting the stage for analyzing the program’s impact on these various patient groups.

### Pain improvement

**FIG. 2.**
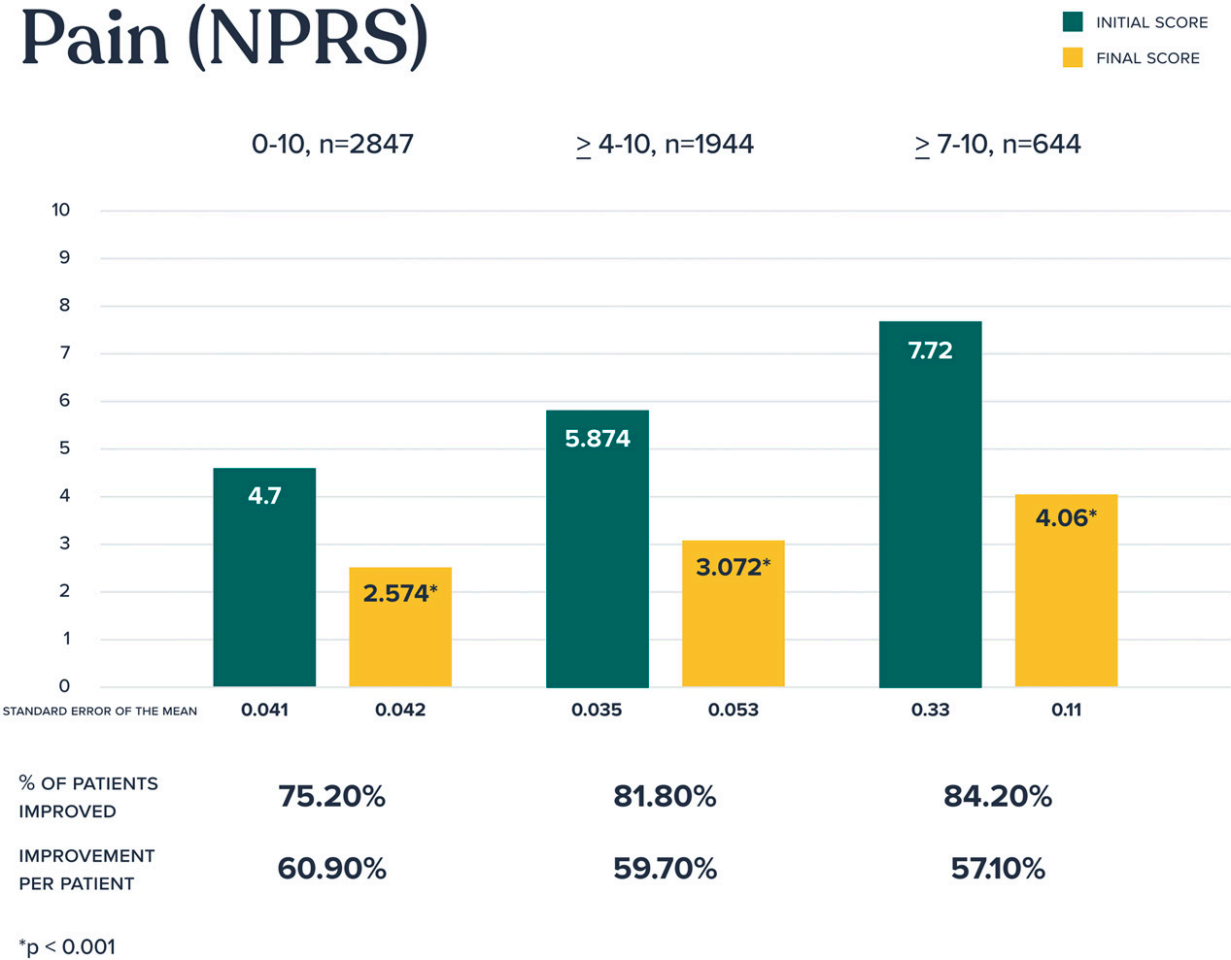
Pain outcomes (Numeric Pain Rating Scale).

Baseline and final numeric pain rating scale scores (scale, 0–10) were available in 2,847 patients (Fig. 2). Statistically significant improvement in pain scores were seen across all groups analyzed with 75.2% of the overall population experiencing clinical improvement in pain. For those with moderate to severe pain (defined as a score of 4 or higher), 81.8% experienced clinical pain improvement, and for the most severely affected group, those with severe pain (defined as a score of 7 or higher), an impressive 84.2% experienced pain improvement. For patients who experienced pain improvement, the mean percentage of pain improvement per patient was approximately 60%.

To explore the relationship between number of clinical visits and pain reduction, a Spearman rank-order correlation was performed, showing a statistically significant, although modest, negative correlation (ρ = –0.15, *p* < 0.001) suggesting that patients who attended more clinical visits tended to experience greater reductions in pain.

Further analysis showed that pain reduction was not significantly correlated with sex (ρ = −0.009, *p* = 0.63) or age (ρ = 0.001, *p* = 0.97). However, as pain levels decreased, there was a statistically significant improvement in functional status, as measured by the SANE score (ρ = −0.28, *p* < 0.001).

To evaluate whether pain reduction varied by body region, a Kruskal–Wallis test was conducted. This test revealed a *p*-value of 0.056. Given this near-significant result, we proceeded with pairwise comparisons to explore regional differences. Although some comparisons showed nominal significance, none held up after applying the Bonferroni correction for multiple testing. This lack of statistical significance may be attributed to limited power in certain body part categories, particularly those with smaller sample sizes, such as the lower extremities.

The correlation between pain improvement and the severity or chronicity of symptoms was evaluated. A weak positive correlation (ρ = 0.077, *p* < 0.001) was observed, suggesting that the severity of symptoms has only a limited impact on the degree of pain reduction.

### Functional improvement

**FIG. 3.**
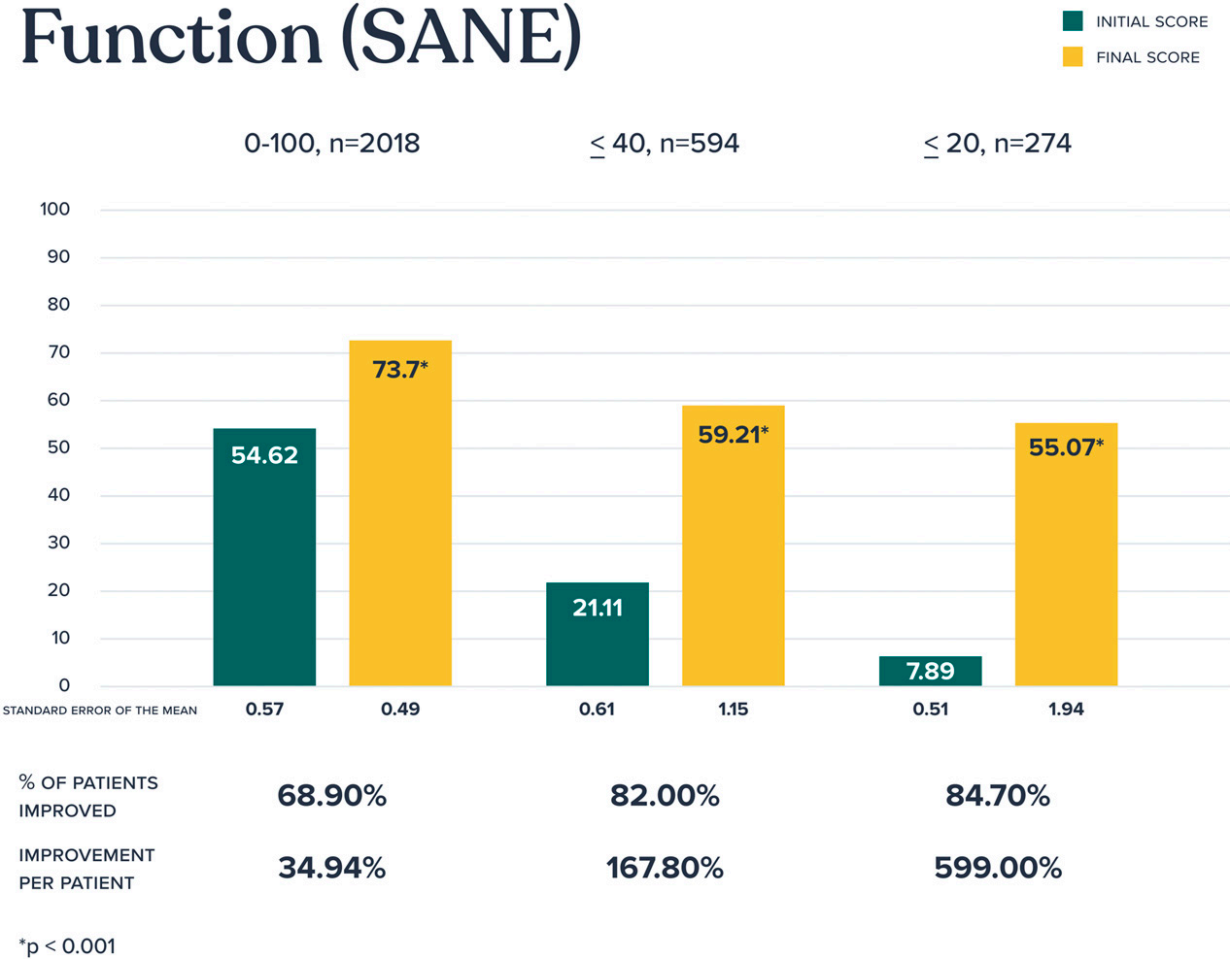
Functional outcomes (Single Assessment Numeric Evaluation).

Baseline and final SANE scores (scale, 0–100) were recorded in 2,018 patients (Fig. 3). Statistically significant improvement in function were seen across all groups analyzed with 68.9% of the overall population experiencing functional improvement. For those with moderate to severe functional compromise (defined as a SANE score of 40 or less), 82.0% experienced improvement, and for the most severely affected group, those with severe functional compromise (defined as a score of 20 or less), an impressive 84.7% experience dramatic functional improvement. For patients with improved function, the mean percentage of improvement per patient was most pronounced for those with moderate to severe functional compromise (167.8%) and those with severe compromise (599.0%).

SANE improvements were not correlated with sex (ρ = −0.008, *p* = 0.73) or age (ρ = −0.004, *p* = 0.86). However, a significant negative correlation (ρ = −0.28, *p* < 0.001) was found between SANE improvements and pain, further reinforcing the positive impact of the program on both functional and pain outcomes.

Further analysis revealed a modest yet statistically significant positive correlation between the number of clinical visits and improvements in SANE functional outcomes. A Spearman rank-order correlation of (ρ = 0.18, *p* < 0.001) indicates that patients who engaged more in clinical visits tended to experience greater functional improvements.

In assessing whether SANE score improvements differed across body regions, a Kruskal–Wallis test was employed to accommodate the non-parametric distribution of the data. The test yielded a statistically significant result (*p* = 0.043), indicating that functional improvement varied by body parts. Post hoc pairwise comparisons revealed nominal significance in some regions, but none remained significant after applying the Bonferroni correction for multiple comparisons. However, the comparison between the wrist/hand/finger group and the back approached significance (adjusted *p* = 0.071), with the back group showing greater improvements in the SANE scores. This result is clinically intuitive, as functional limitations stemming from back pain often impact a broader range of daily activities, potentially leading to more substantial recovery following intervention.

The correlation between SANE improvements and the severity or chronicity of symptoms was assessed. A weak negative correlation (ρ = −0.059, *p* = 0.011) was observed, suggesting that the initial severity or chronicity of symptoms had only a minimal impact on functional improvement.

### Mental health improvement

The study cohort demonstrated minimal anxiety at baseline based on the GAD-2 score. A total of 1,446 patients had a mean starting score of 1.14 ± 0.039, and slightly improved mean final score of 0.98 ± 0.036 (*p* < 0.001). Positive screening for generalized anxiety disorder (score of 3 or higher) occurred in 185 patients (12.8%) with the average starting score of 4.24 ± 0.085, which significantly decreased to 2.60 ± 0.14 (*p* < 0.001).

The study cohort demonstrated minimal depressive symptoms at baseline based on the PHQ-2 questionnaire. A total of 1,069 patients had a mean starting score of 0.82 ± 0.037, and mean final score of 0.74 ± 0.037, indicating that the population maintained a stable status. Positive PHQ-2 screening for a potential major depressive disorder (score of 3 or higher) occurred in 93 patients (8.7%) with a mean starting score of 3.77 ± 0.098 which significantly decreased to 2.43 ± 0.20 (*p* < 0.001). This substantial reduction reflects a 35.6% improvement in this subgroup.

## Discussion

Effective management of a population of MSK patients requires recognition of the diversity and complexity of clinical conditions and patients’ symptoms. Point solutions which address only one aspect of MSK care are likely to be less effective than holistic models across populations. Our study highlights the effectiveness of a digital physician-led MSK IPU in improving pain and physical function based on a robust sample size of 2,851 patients and rigorous statistical analyses to elucidate clinical improvements across patient subgroups stratified by severity of pain and functional limitations.

Primary findings in our study include both significant improvement is pain and function across a diverse MSK population. Overall, 75.2% of our patients had pain improvement and 68.9% had functional improvement. In our analysis of patients with greater severity of pain (score of 7 or higher), our model supported impressive clinical improvements: 84.2% of patients experienced meaningful pain relief (average pain score change from 7.7 to 4.1). Given that most patients seek evaluation for MSK concerns with a SANE score in the mid 50s,^25^ the functional improvement of patients in our model with a SANE score of 20 or less is impressive (mean SANE score improved 599% from 7.9 to 55.1). These improvements are not only statistically but clinically significant.

Many factors will influence the clinical improvement in patients. While the correlation was weak, our data support the notion that increased clinical visits may contribute to better patient-reported functional outcomes. A weak negative correlation was seen between SANE scores and severity or chronicity of symptoms, suggesting that the initial severity or chronicity of symptoms had only a minimal impact on functional improvement. This finding underscores the importance of other factors in predicting recovery, suggesting that functional recovery may not be solely dependent on the baseline severity of symptoms but rather on a complex interplay of clinical, personal, and environmental factors. We believe our IPU approach which incorporates behavioral health and lifestyle modification may have contributed to the impressive functional improvement we observed in our most severely compromised group of patients.

Patients with MSK conditions often have co-existing mental health conditions such as anxiety and depression. In our cohort, 12.6% of patients had a positive screen for generalized anxiety disorder and 8.4% for a major depressive disorder. Our digital IPU provides cognitive behavioral therapy support to these patients and to communicate mental health concerns with patients’ primary care physicians. While the number of patients with positive screens is relatively small, we did see statistically significant improvement in these elevated scores. As mental health issues can negatively impact treatment and clinical outcomes for MSK conditions,^26,27^ recognizing them and providing resources to patients to address mental health concerns are essential to optimize care.

Overall, these results demonstrate the clear effectiveness of a digital MSK IPU in alleviating pain, improving function, and positively impacting anxiety and depression across various levels of severity. We believe that these outcomes are the result of our physician-led teams providing continuous clinical oversight, personalized care planning, and adaptive interventions that respond dynamically to patient progress and barriers.

### Limitations

Our study has several limitations. Rates of imaging and referral to in-person services (surgeon, rheumatologist, physical therapist) were not included in this analysis but will be performed in a future study. Historically, our IPU has a low rate of surgical referral; <3% in two published cohorts.^28,29^

Our analysis did not discern patients who developed a second MSK condition and returned to us for treatment of additional conditions. In this scenario, the pain and functional scores at most recent follow-up could be lower than the baseline scores of the original condition, artificially showing lower outcomes. Future research will identify these patients and support more complex data analysis.

While we set an inclusion criteria for a least three clinical visits for this study, not all patients elected to continue clinical care with some preferring to convert to self-management once they started to make progress. Specifically, 9.5% of patients had 2 weeks or less of clinician services. These patients also included those who were referred or preferred in-person services shortly after their IPU evaluation and those who were determined by the clinicians to be inappropriate for digital care (e.g., significant fall risk needing in-person supervision).

## Conclusion

Integrating MSK expert physicians and nurse practitioners with physical therapists, health coaches, and registered dietitians in an evidence-based, biopsychosocial digital IPU is an effective model for treating patients across the broad spectrum of MSK conditions including patients with severe symptoms. We believe our experience represents the first published results of a digital MSK IPU. Our findings advocate for widespread adoption of specialty physician-led, team-based MSK care to meet the growing demand for impactful, efficient, and accessible MSK care in the increasingly digital age.

## Abbreviations Used

ANOVA: Analysis of variance
GAD-2: Generalized Anxiety Disorder Scale-2
IPU: Integrated practice unit
MD: Medical Doctor
MSK: Musculoskeletal
NP: Nurse practitioner
PHQ-2: Patient Health Questionnaire-2
PT: Physical therapist
PTA: Physical therapy assistant
SANE: Single assessment numeric evaluation
